# Exosomes in review: A new frontier in CAR-T cell therapies

**DOI:** 10.1016/j.neo.2025.101147

**Published:** 2025-03-03

**Authors:** John S. Wang, Samuel J. Schellenberg, Athena Demeros, Adam Y. Lin

**Affiliations:** a Northwestern University, Feinberg School of Medicine, Department of Medicine, Chicago, IL, USA; b Northwestern University, Evanston, IL, USA; c Northwestern University, Feinberg School of Medicine, Department of Medicine, Division of Oncology, Chicago, IL, USA; d Robert H Lurie Comprehensive Cancer Center of Northwestern University, Chicago, IL, USA

**Keywords:** Exosome, Chimeric antigen T cell therapy, Immunotherapy

## Abstract

Exosomes are extracellular vehicles that facilitate intra-cellular communication via transport of critical proteins and genetic material. Every exosome is intrinsically reflective of the cell from which it was derived and can even mimic effector functions of their parent cells. In recent years, with the success of CAR-T therapies, there has been growing interest in characterizing exosomes derived from CAR-T cells. CAR exosomes contain the same cytotoxic granules as their parent cells and have demonstrated significant anti-tumor activity *in vitro* and in animal models. Moreover, infusion of CAR exosomes in animal models did not generate cytokine release syndrome. Conversely, there are also novel bispecific antibodies which target tumor-derived exosomes in hopes of derailing immunosuppressive pathways mediated by exosomes produced from malignant cells. The two most promising examples include (a) BsE CD73 x EpCAM which binds and inhibits exosomal CD73 to suppress production of immunosuppressant adenosine and (b) BsE CD3 x PD-L1 which targets exosomal PD-L1 within the tumor microenvironment to guide cytotoxic T-cells towards tumor cells. As our understanding of exosome biology continues to evolve, opportunities for advances in cellular therapies will grow in tandem.

## Engineered T cells changed the landscape of hematologic malignancy therapy

In recent years, engineered chimeric antigen receptor T-cells (CAR-T) therapy has been an area of rapid development brimming with potential applications in malignant hematology [1]. Since the first patient, Emily Whitehead, received CAR-T cells in 2012 with a durable remission to date, the field has grown to encompass multiple approved products for B-cell lymphomas, multiple myeloma, and acute lymphoblastic lymphoma [2]. For large B-cell lymphomas, lisocabtagene maraleucel (liso-cel) (TRANSCEND) and axicabtagene ciloleucel (axi-cel) (ZUMA-1) have shown impressive results as a third-line therapy, with some patients even possibly cured [[3], [4], [5], [6]]. Real-world experience showed that the benefits of CAR-T therapy seen in the trials are persistent even on increasingly distant follow-up from initial treatment [7]. With updated results from the second line phase III trial (TRANSFORM) with liso-cel presented at ASCO 2024, the 3-year follow-up showed sustained improved outcomes of CAR-T over standard of care (SOC) compared to the primary analysis (overall response rate (ORR) 87 %, complete response (CR) 74 %, median event-free survival (EFS) 29.5mo, and 3-year overall survival (OS) 62.8 % for the CAR T arm) [8,9]. Since the approval of the first CAR-T cell therapy tisagenlecleucel in 2017, they have found a niche in the treatment of hematologic malignancies, initially only for relapsed and refractory disease, but now also as first-line agents as clinical trials continue to carve out new roles for cellular therapies.[1]

Understanding methods to improve T-cell populations and eliminate harmful agents to cellular fitness is an evolving field of research. For example, treatment with bendamustine leads to significant T-cell depletion before apheresis and results in markedly reduced CAR-T cell efficacy (ORR 53 % vs 72 %, PFS 3.1 vs 6.2 months, OS 10.3 vs 23.5 months, with or without bendamustine exposure, respectively) [10]. Low absolute lymphocyte count (ALC) prior to CAR-T therapy and low ALC after CAR-T therapy are also both correlated with worse outcomes [11,12]. Not only the quantity of T cells is important for the response to CAR-T therapy, but also the fitness and phenotype of the T cells can impact the effectiveness of these treatments [[13], [14], [15], [16]]. As we continuously deepen our understanding of T-cell biology, the topic of T-cell exosomes is one of the newest entities to garner scientific focus in pursuit of enhancing cellular therapies.

## What is an exosome

**Fig. 1:**
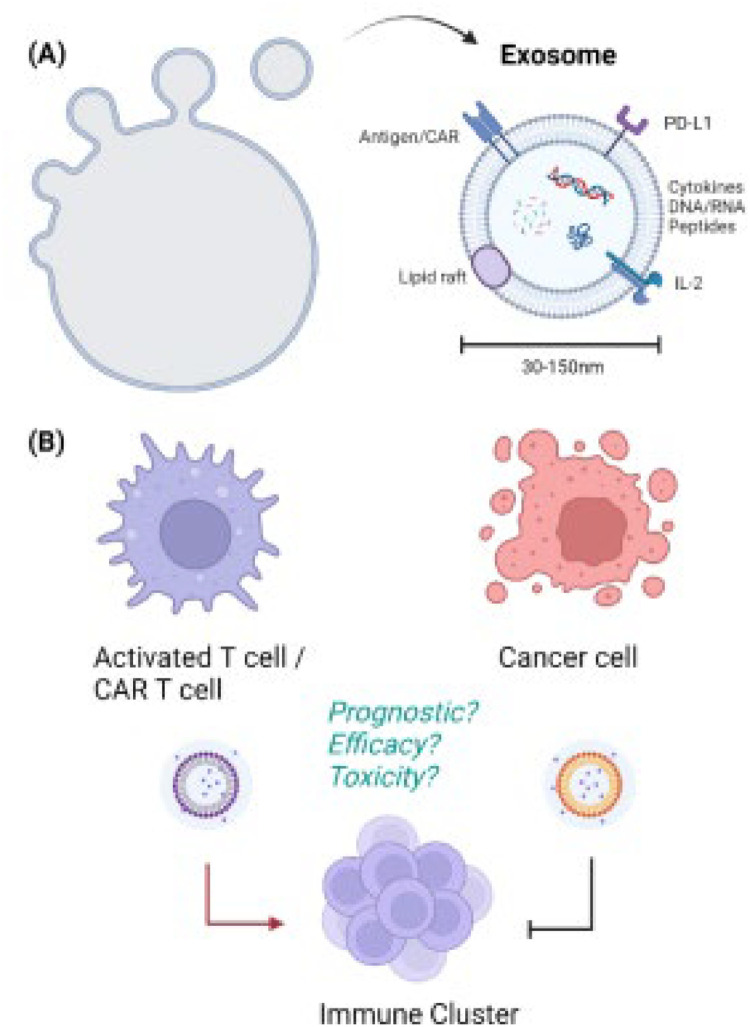
Exosomes and T cell cancer immunity. (A) Exosomes are small vesicles that play a crucial role in cellular communication by transporting proteins, nucleic acids, and surface proteins including chimeric antigen receptor (CAR), program cell death ligand 1 (PDL1), or interleukins (IL). (B) Both CAR exosomes and tumor-derived exosomes may offer advantages in enhancing T-cell therapies.

Extracellular vesicles (EVs) are a heterogenous group of membrane-bound structures that play an integral role in cellular communication by exchanging signaling proteins, genetic material, and other cargo [17]. EVs are derived from the endocytic compartment or the cell membrane directly allowing them to be highly stable in body fluids due to lipid-rich membranes enriched with cholesterol, sphingomyelin, and ceramides [18] (Fig. 1A). EVs are generally classified into two major categories: ectosomes and exosomes. Ectosomes are between 50 nm and 1 μm in diameter and bud directly from the plasma membrane. Exosomes, on the other hand, are smaller, around 30-150 nm in diameter, and come from the cellular endosome before being released into the extracellular space to aid in intracellular communication [17]. Exosomes specialize in cell-to-cell transport of proteins, nucleic acids, and metabolites. In some instances, exosomes can serve as a source of peptides for major histocompatibility complexes that serve in antigen presentation and priming of the adaptive immune system [18]. A better understanding of their biogenesis and function would open numerous avenues of further research in harnessing exosomes as a resource for treating various autoimmune and oncologic diseases.

Exosomes are intraluminal vesicles contained within multivesicular bodies; the intraluminal vesicles are only dubbed “exosomes” upon fusion of the multivesicular bodies’ membrane with the plasma membrane during exocytosis to release the intraluminal vesicles into the extracellular space. Multivesicular bodies are generated when extracellular constituents such as proteins, metabolites, and other small molecules are endocytosed into a cell resulting in the constituents being enveloped by a portion of the cell membrane containing cell surface proteins of the native cell [17]. The trans-Golgi and endoplasmic reticulum complex processes these new membrane-bound buds into an early sorting endosome. Within these early-sorting endosomes, another round of plasma invagination occurs to generate multiple buds of intraluminal vesicles (future exosomes) within the membrane of the early-sorting endosome which then develops into a multivesicular body. The final result is a multivesicular body whose outer membrane encapsulates multiple smaller intraluminal vesicles that can be released into the extracellular space upon fusion of the multivesicular body's membrane with the cell's plasma membrane during exocytosis [17]. The exosomes released can contain both extracellular constituents that the native cell acquired via endocytosis and native cell membrane proteins that were invaginated from the outer cell membrane during the process of endocytosis. Therefore, the components of an individual exosome are wildly heterogenous depending on their cell of origin.

## T-cell biology and T-cell exosomes

Broadly, all T-cells are subdivided into two main subgroups: (1) CD4^+^ helper T-cells which play an ancillary role in supporting the function of other immune cells and (2) CD8^+^ cytotoxic T-cells, including cytotoxic lymphocytes (CTLs) which take a more active role in killing their targets [19]. T-cell development occurs in the thymus wherein precursor cells undergo multiple rounds of selection and lineage differentiation after arrival from the bone marrow as a naïve cell [20]. After losing their multipotent potential via the Notch signaling pathway, the newly committed thymocytes undergo T-Cell Receptor (TCR) recombination under the control of recombination-activating genes (RAG) which break, re-arrange, and repair the TCR-alpha and TCR-beta genes and dictate the receptor's function [21,22]. Next, the thymocytes undergo a process of positive selection mediated by cortical thymic epithelial cells which specifically identify thymocytes whose TCRs have the ability to bind Class I or Class II MHC plus self-peptides expressed by the cortical thymic epithelial cells [23]. The positive selection process develops T-cells into MHC-restricted CD4^+^ or CD8^+^ cells [19,23]. Subsequently, the thymocytes undergo a negative selection process that removes cells with the propensity to recognize and respond to self-antigens [24]. The final result is a CD4^+^ or CD8^+^ *T*-cell population meticulously developed to carry out their respective roles for our adaptive immune system.

CD4^+^ *T*-cells activate upon interaction with the antigen-MHC II complex presented by antigen-presenting cells (APCs) and go on to play crucial roles in modulating our immune system via activation of B-lymphocytes, cytotoxic CD8^+^ *T*-cells, and even other non-immune cells [25]. In recent years, there has been increased interest in how CD4^+^ *T*-cells and their derivative exosomes interact with other immune cells as we explore potential mechanisms for harnessing the adaptive immune systems against autoimmune, anti-viral, and anti-tumor activity [26]. Hong et al. found that exosomes from interleukin-2 (IL-2) activated CD4^+^ *T*-cells could be used to re-activate latent HIV in other CD4+ *T*-cells so that previously latently infected cells could be more easily found and killed by CTLs in a macaque animal model [27]. Exosomes from CD4^+^ *T*-cells can also prime dendritic cells via the transfer of genomic and mitochondrial DNA to activate anti-viral responses within the dendritic cells (e.g. inducing expression of interferon-regulated genes) to make them less susceptible to future viral infections [28]. Building off of these foundational concepts, Jung et al. modified Jurkat T-cells to produce an IL-2 on their plasma membranes resulting in the release of IL-2 carrying exosomes containing micro-RNA (miRNA). The expression of miRNA was increased due to the autocrine effect of IL-2 on the plasma membrane of the Jurkat T-cells [29]. These miRNA-containing exosomes were shown to have direct anti-tumor activity while augmenting other immune cells in the tumor microenvironment.

CD8^+^ CTLs are the backbone of the adaptive immune system and are critical effectors in removing intracellular pathogens and facilitating anti-tumor surveillance [30]. CTL activation happens after interacting with a tumor antigen-MHC I complex; this presentation leads to priming of CD8^+^ *T*-cells against the tumor cells. Furthermore, the field has gradually elucidated the role of CTL-derived exosomes in direct tumor killing. Seo et al. found that activated CTLs in a murine model release cytotoxic exosomes that deplete the mesenchymal tumor cells necessary for progressive tumor invasion and metastasis pathways.[31] Thus, CTLs facilitate both direct cytotoxic activity against tumor cells, and indirect disruption of tumor proliferation through secretion of exosomes. Li et al. manufactured a mouse T-cell line to over-express PD-1 so that any derivative exosomes would also display high levels of PD-1 [32]. These exosomes prevent tumor-intrinsic PD-L1 from binding PD-1 on CTLs, thus avoiding inhibition of the immune cells.

## CAR-T cell therapies and CAR exosomes

CAR T cells contain engineered receptors comprised of an extracellular-binding domain as a single-chain variable fragment primed to recognize tumor antigens (CD19 for lymphoma and BCMA for myeloma) and an intracellular component responsible for T cell activation [33]. As with any other T-cell, the manufactured CAR-T cells also secrete exosomes amongst other extracellular vesicles. Similar to their CTL precursors, CAR-T cells release exosomes that carry their manufactured chimeric antigen receptor on their lipid membrane surfaces in addition to high quantities of cytotoxic molecules [34]. Therein, an exosome with all of the integral capabilities of a CAR-T cell opens a new world of possibilities for oncologic therapies, especially in the sphere of malignant hematology.

CAR exosomes carry CAR proteins at the same surface concentration found on their progenitor CAR-T cells. Fig. 1B under electron microscopy, all purified exosomes were found to be homogenous in size (∼80 nm in diameter) with additional expression of MHC I proteins, CD3, CXCR4, and CD57. Notably, PD-1 expression was undetectable on exosomes, which confers immunity from tumor cell PD-L1 pro-apoptotic signaling mechanisms [34]. The nanoscale diameter of the exosome confers size-specific advantages in penetrating the solid tumor microenvironment which has been a historic barrier for use of the much larger CAR-T cells [35]. Yang et al. speculated that mesothelin-targeted CAR-T cell exosomes could be used to treat triple-negative breast cancers [36]. Exosomes were generated with human CAR T cells targeting mesothelin-targeted single-chain variable fragments in culture. After confirming that they had the same membrane morphology as their parent cells, exosomes were injected into a murine breast cancer model that showed a dose-dependent effect of exosome administration on tumor growth inhibition. The exosomes passed through the tumor's extracellular matrix effectively and killed target cells via secreting granzyme B and perforin [36]. Moreover, cell-free exosomes are not subject to the same immunosuppressive modifications of T-cells within the tumor microenvironment that has dampened the effectiveness of CAR-T in solid tumors [37].

CAR-T penetration is an important consideration for effectiveness, especially for tumors in the central nervous system (CNS). CAR-T routes of entry are limited to three modalities: (1) post-capillary venules in the perivascular space, (2) extravasation through the choroid plexus, or (3) through super leptomeningeal vessels [38]. All three modalities while feasible are not optimized for CNS delivery from systemically administered CAR-T cells. Therefore, intra-thecal or intra-tumoral administration is preferred amongst the few pediatric clinical Phase I trials for CAR-T therapies in refractory CNS tumors [39]. Due to their small size, exosomes do not share the same difficulties or dependencies on the aforementioned transport mechanisms. Given the inverse relation between substrate size and the ability to cross the blood-brain barrier (BBB), exosomes have already been researched as potential vehicles to deliver pharmacologic agents to the central nervous system [40]. While their mechanism of transport in the BBB is not fully understood, the most commonly posited route is via transcytosis through the endothelial cells that line the barrier [41]. With our current understanding of the BBB, we posit that systemically administered CAR exosomes could penetrate the central nervous system and may enhance CAR-T efficacy. Thus, there could exist a role for CAR exosome therapies where clinical trials are currently assessing the response of refractory pediatric brain tumors to CAR-T cell therapy.

Due to the carrier functionality of all exosomes, CAR exosomes can also be loaded with anticancer drugs to deliver a synergistic therapy that combines the pharmacologic therapy with the natural cytotoxic elements of the CAR exosome.[42] Due to the unique bio-properties of exosomes, this could open novel routes of administration for chemotherapeutic agents that are typically delivered intravenously. For example, CAR exosomes have been used to pilot inhaled delivery of paclitaxel in non-small cell lung cancer mouse model in an attempt to reduce systemic toxicities of treatment; CAR exosomes were derived from a CAR-T cell engineered to target mesothelin on lung cancer cells and paclitaxel was loaded into the CAR-derived exosomes. Inhalation of the exosomes containing paclitaxel reduced tumor size and prolonged survival time in the mouse model.[43]

## CAR exosomes do not provoke cytokine release syndrome

Despite the impressive efficacy of CAR-T therapy, the toxicities can be severe albeit manageable. The main adverse event is cytokine release syndrome (CRS) due to high levels of immune activation [44,45]. On the cellular level, CRS manifests from a storm of inflammatory cytokines from activated lymphoid (B-cells, T-cells, and NK cells) or myeloid cells (monocytes, dendritic cells, and macrophages) [44]. Clinically, it can manifest as fevers, hypoxia, and hypotension with severe cases requiring critical care level monitoring in the setting of vasopressor or intubation requirement [46]. Rates of CRS secondary to CAR-T cell therapy vary greatly depending on the type of tumor being treated, the active tumor burden, and the specific CAR-T cell infused. For example, amongst large B-cell lymphoma patients, CRS is estimated to occur in anywhere from 28 to 35 % treated with CAR-T therapies. Grade 3 to 4 CRS occurs in anywhere from 1 to 22 % of patients treated with CAR-T therapies. The range varies between the commercial CAR-T product infused [4,47,48].

On the other hand, CAR exosomes do not generate the inflammatory cytokines underlying CRS [34]. CAR T-cells and CAR exosomes were infused into mouse breast cancer models to compare rates of cytokine release *in vivo*. CAR-T cells were administered in escalating doses, and a dose-dependent effect was observed for subdued behavior, weight loss, reduced mobility, and piloerection. Serial blood analyses throughout treatment showed elevated levels of interferon-γ, interleukin-2, and interleukin-6. In comparison, CAR exosomes administered in escalating doses yielded no alterations in behavior or recorded weight. There was also no cytokine elevation detected on serial blood analyses of CAR exosome treated mice [34]. Yang et al. also treated mouse models with their anti-mesothelin variant of CAR exosome at an escalating dosing gradient, and even at maximum exosome dosing, no toxicities were recorded. The mice exhibited no changes in their body weight and no pathologic change in their heart, liver, or splenic tissue after treatment [36].

## Examining CAR exosomes in relation to ICANS

Another notable adverse effect of CAR-T cell therapies is the development of immune cell-associated neurotoxicity syndrome (ICANS). Its mechanism is not perfectly understood, but it is posited to be an immunotherapy-induced inflammatory response that disrupts the blood-brain barrier allowing increased infiltration of lymphocytes, monocytes, and cytokines [49,50]. Collectively, this leads to endothelial activation, systemic capillary leakage, and thrombotic microangiopathy which all further compromise the integrity of the blood-brain barrier [51]. Early presenting symptoms of ICANS include expressive aphasia, tremors, dysgraphia and fatigue, and can then quickly evolve into more severe symptoms of global aphasia, seizures, and coma.[50] Most cases occur between three to ten days after CAR-T cell infusion [49]. Although ICANS frequently develops in patients with CRS, some patients experience ICANS in isolation [49]. While there has been initial data showing that CAR exosomes do not provoke CRS, there are no studies to date to support the same observation in ICANS.

On the contrary, recent studies suggest a role for CAR exosomes in the development of ICANS. Storci et al. measured CAR exosome levels after infusion of CD-19 targeted CAR-T cells in patients with relapsed or refractory B-cell lymphomas [52]. Exosomes were measurable as early as one hour post-infusion and the median level of CAR exosomes was higher in patients who would later develop ICANS versus in those who did not. CAR exosomes were cultured with human-derived progenitor and mature neurons which provoked higher levels of Enolase-2+ nanoparticle release, a marker of neuronal stress. Subsequently, Enolase-2+ levels were elevated in patients who developed ICANS after CAR-T therapy when compared to patients who did not. In totality, the data suggests that CAR exosome levels may be used as an early predictor of ICANS development and further work is needed to elucidate the role of CAR exosome secretion in the pathogenesis of ICANS [52]. Felice et al. tested a similar hypothesis with results that support the same conclusion; they measured CAR exosome levels in B-cell lymphoma patients who had received CAR-T cell therapies and found that serum concentration levels greater than 187.5 CAR EV/μl predicted ICANS onset with a specificity of 83 % and sensitivity of 100 % [53] (Table 1).

**Table 1:** Comparison of CAR-T cells versus CAR exosomes.

	CAR-T Cells	CAR Exosomes
Target Selectivity	+	+
Size	+	–
Risk of CRS	+	–
Risk of ICANS	+	++
CNS Penetration	+	+++
Sensitivity to PD-L1	+	–
Difficulty to Manufacture	+	+++
FDA Approval	+	–

## Difficulties in manufacturing exosomes

In order to use exosomes in clinical settings, a reliable, rapid, and large-scale synthesis method is required. The state of our current technology has marked difficulty isolating and purifying in meaningful quantities. In the literature, different methods include immunoaffinity capture, size exclusion chromatography, ultrafiltration, and ultracentrifugation [54]. Immunoaffinity capture uses monoclonal antibodies to locate and capture exosomes thus producing a high-purity product, but it is not a sustainable or scalable method due to high expenses and low yields [55]. Size exclusion chromatography can economically separate nanoparticles based on the estimated range of an exosome's diameter, but is subject to lipoprotein contaminants of similar size [56]. Ultrafiltration offers a quick, cost-effective, and technically viable methodology, but struggles to yield a pure product as it can only separate exosomes from protein contaminants [57]. Ultracentrifugation covers desired criteria of high purity, low cost, and high yields, but there is notable concern about the process damaging the isolate's cellular structure [58]. Given the benefits and drawbacks of each different method, the optimal answer would logically be a combination protocol to leverage individual strengths. A combination method of ultracentrifugation, size exclusion chromatography, and repeat centrifugation through a filter improved final yield concentrations [59]. The combination yields a high quantity of pure exosomes that were confirmed to be intact structurally via electron microscopy. However, as with any combination method, the protocol is more time-intensive and expensive than any individual method alone. Fortunately, exosomes can be stored post-production, but additional attention is required to fully elucidate the degree of particle loss and purity reduction over time [42,60].

## Tumor-derived exosomes

Similar to T-cells, malignant cells can also secrete exosomes of their own which have been implicated in both the development of the tumor microenvironment and immune surveillance evasion. The molecular signature of these tumor-derived exosomes (TDEs) mimics the surface of their parent tumor cells which helps distinguish TDEs from exosomes produced by non-malignant cells [61]. For example, hepatocellular carcinoma cells can secrete exosomes containing a high level of miR-1247-3p. This mIR is known to convert normal fibroblasts via the β1-integrin-NF-κB signaling pathway into cancer-associated fibroblasts responsible for secreting pro-inflammatory cytokines such as IL-6 and IL-8 [62]. Given the level of mimicry to parent tumor cells, exosome-based liquid biopsy is a novel topic that has garnered research interest in recent years [63,64]. As tumor-derived exosomes freely circulate the peripheral blood, their cargoes can be isolated and sequenced for matching against pre-identified tumor molecular profiles. This is especially helpful in cases where tissue biopsy is technically difficult or high-risk. For example, in hepatocellular cancer, biomarkers such as miRNA-92b carried in TDEs have been linked to disease recurrence in post-liver transplant patients [65]. If able to be effectively isolated and sequenced from serum TDEs, it could serve as an useful biomarker in disease surveillance.

Regarding immune surveillance, certain solid tumor cells (e.g. bladder, breast, GI, and prostate) can secrete extracellular adenosine to actively suppress local anti-tumor immune activity. The pathway of adenosine generation is dependent on tumor-derived exosomes expressing CD39 and CD73 jumpstarting the conversion of 5′AMP to adenosine which is toxic to immune cells [66,67]. In this process, CD73 is especially critical as it is the rate-limiting step for the enzymatic dephosphorylation of 5′AMP into adenosine that diffuses into the tumor microenvironment to facilitate inhibition of local immune cells [68]. There is already scientific interest in exploring CD73 as a putative target in cancer treatment. Bendell et al. conducted the first phase I clinical trial proving a tolerable safety profile in patients with advanced solid tumors for combination therapy with oleclumab, an anti-CD73 human IgG1λ monoclonal antibody, and durvalumab, an anti-PD-L1 monoclonal antibody [69]. However, oleclumab was designed to target CD73 expressed on tumor cells and does not effectively inhibit CD73 carried on tumor-derived exosomes. To this end, a novel bispecific antibody (CD73xEpCAM) was developed to bind both CD73 and EpCAM, a common exosome surface marker and has demonstrated potent inhibition of CD73^+^/EpCAM^+^ carcinoma cell lines *in vitro* [68].

**Fig. 2:**
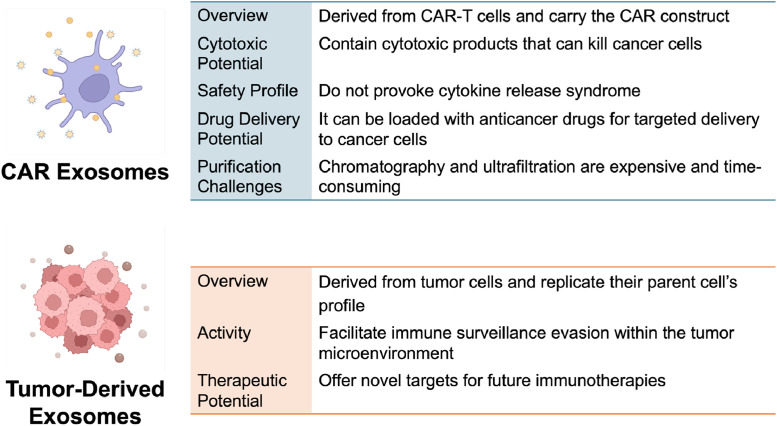
Key characteristics defining CAR exosomes and Tumor-Derived Exosomes.

Another mechanism by which malignant cells could evade immune surveillance is via the secretion of tumor-derived exosomes containing PD-L1 [70]. Levels of exosomal PD-L1 expression on exosomes have direct correlation with disease progression in multiple tumors types across the spectrum of melanoma, HNSCC, NSCLC, and diffuse large B-cell lymphomas [71]. Exosomal PD-L1 plays an important role in the tumor microenvironment by conveying a regulatory signal to induce T-cell anergy, exhaustion, and eventual apoptosis [72,73]. In a clever reversal of roles, Cho et al. demonstrated that a bispecific T-cell antibody designed to bind CD3 x PD-L1 could harness surrounding exosomal PD-L1 as a migratory beacon to increase infiltration of CD8+ cytotoxic T-cells into solid tumor models *in vitro*. The phenomenon of improved T-cell infiltration into tumor tissue was abolished by the application of an exosome-secretion inhibitor thus providing proof of concept that it was exosomal PD-L1 mediating their observation [74]. More research is needed to better elucidate the exact role of tumor-derived exosomes within the tumor microenvironment. Still, the existing body of work has already belied their potential significance and begun to capitalize on our progressive understanding (Fig. 2).

## Clinical conclusions and future challenges

As our understanding of exosomes has significantly advanced over the last decade, there are new applications for exosomes to change the landscape of cancer therapy. Exosomes are fascinating as they host different proteins and genetic material that reflect the parent cell from which they were derived. Given the impact of CAR-T cell therapies in oncology, the question arises of whether CAR exosomes could provide benefit. In the emerging literature on the topic, exosomes contain cytotoxic granules capable of killing tumor cells *in vivo* and potentially offer a safer toxicity profile by not provoking cytokine release syndrome. The physically smaller size of an exosome also facilitates increased penetration of the blood-brain barrier and deeper infiltration of the extracellular matrix in solid tumors [35,40]. Moreover, CAR exosomes still retain the their properties as an extracellular vehicle and can be loaded with chemotherapeutic agents opening novel modalities of targeted drug administration [43].

Unfortunately, there still remain many challenges to clinical application of exosome-based therapies. For example, optimization of the purification process is a rate-limiting step towards offering commercial exosome products. Immunoaffinity capture, size exclusion chromatography, ultrafiltration, and ultracentrifugation have their respective strengths and weaknesses. Although combining these methods improve both the yield and purity of the final product, the process remains both economically demanding and time-consuming. There are also certain aspects of exosome biology that might complicate translation into clinical therapies. As discussed earlier, the smaller size of an exosome compared to its parent cell allows for better CNS penetration, but there are concerns that CAR exosomes could provoke higher rates of ICANS than CAR-T cells [52]. Advancements in radio-labeling, PET, and SPECT imaging has made it easier to track infused exosomes, yet further work is needed to fully characterize *in vivo* behavior of exosomes before therapies can be broadly adopted in clinical practice [75,76].

Meanwhile, tumor-derived exosomes play their own role within the tumor microenvironment by acting as an extension of their parent cell and facilitating immune surveillance evasion. Due to their ability to enter systemic circulation, TDEs are a potential target for liquid biopsy and sequencing of their cellular cargo can help detect or monitor disease. Integration may become feasible in the near future pending improvement of the exosome purification process on a commercial scale [63]. Research on TDE-mediated immunosuppression has also improved our understanding of the tumor microenvironment and opened new options for targeted therapies directed against exosome-mediated immune surveillance [69,74]. With the existing advances in exosome biology, it is only a function of time before the progress seen *in vitro* converts into full-fledged clinical trials with the hope of introducing new exosome-based therapies to the patients who need them most.

## CRediT authorship contribution statement

**John S. Wang:** Investigation, Writing – original draft, Writing – review & editing. **Samuel J. Schellenberg:** Writing – review & editing. **Athena Demeros:** Visualization, Writing – original draft. **Adam Y. Lin:** Conceptualization, Investigation, Methodology, Supervision, Visualization, Writing – original draft, Writing – review & editing.

## Declaration of competing interest

The authors declare that they have no known competing financial interests or personal relationships that could have appeared to influence the work reported in this paper.
